# Validation of educational material for the care of people with intestinal stoma*

**DOI:** 10.1590/1518-8345.3179.3269

**Published:** 2020-05-11

**Authors:** Julliana Fernandes de Sena, Isabelle Pereira da Silva, Silvia Kalyma Paiva Lucena, Adriana Catarina de Souza Oliveira, Isabelle Katherinne Fernandes Costa

**Affiliations:** 1 Universidade Federal do Rio Grande do Norte, Departamento de Enfermagem, Natal, RN, Brazil.; 2 Faculdade de Enfermagem, Universidade Católica de Murcia, Murcia, MU, Spain.

**Keywords:** Ostomy, Self-Care, Educational Technology, Health education, Nursing Care, Validation Studies, Estomia, Autocuidado, Tecnologia Educativa, Educação em Saúde, Cuidados de Enfermagem, Estudos de Validação, Ostomía, Autocuidado, Tecnología Educativa, Educación en Salud, Cuidados de Enfermería, Estudios de Validación

## Abstract

**Objective:**

to validate an educational booklet for people with intestinal stoma as a technological resource in the teaching of self-care.

**Method:**

a methodological research for the construction and validation of an educational booklet by nine expert judges and 25 people with stomas. The agreement index of at least 80% was considered to guarantee the validation of the material.

**Results:**

regarding the objectives of the booklet, all the judges evaluated the items as “adequate” or “totally adequate”, with a content validity index of 1.00. Regarding the structure and presentation of the booklet, the total index was 0.84. Regarding relevance, the total was 0.97 and the general index of the educational booklet was 0.89, confirming the validation with the judges. All items of the organization, writing style, appearance and motivation of the material were considered as validated by the target audience, reaching a total agreement index of 0.99.

**Conclusion:**

in the context of health education, the booklet was considered valid and suitable for the care of people with intestinal stoma, and can be used in teaching, research, extension and care for people with intestinal stoma.

## Resumo

**Objetivo:**

validar uma cartilha educativa para pessoas com estomias intestinais como recurso tecnológico no ensino do autocuidado.

**Método:**

pesquisa metodológica para a construção e validação de cartilha educativa por nove juízes especialistas e 25 pessoas com estomias. Foi considerado o índice de concordância de, no mínimo, 80% para se garantir a validação do material.

**Resultados:**

quanto aos objetivos da cartilha, todos os juízes avaliaram os itens como “adequado” ou “totalmente adequado”, com índice de validade de conteúdo de 1,00. Com relação à estrutura e apresentação da cartilha, o índice total foi de 0,84. No quesito relevância, o total foi de 0,97 e o índice geral da cartilha educativa foi de 0,89, confirmando a validação junto aos juízes. Todos os itens da organização, estilo da escrita, aparência e motivação do material foram considerados validados pelo público-alvo, atingindo índice de concordância total de 0,99.

**Conclusão:**

no contexto da educação em saúde, a cartilha foi considerada válida e adequada para o cuidado das pessoas com estomias intestinais, podendo ser utilizada em ambientes de ensino, pesquisa, extensão e no cuidado à pessoa com estomia intestinal.

**Objetivo:**

validar una guía educativa para personas con ostomías intestinales, como recurso tecnológico en la enseñanza del autocuidado.

**Método:**

investigación metodológica para la construcción y validación de una guía educativa por nueve jueces especialistas y 25 personas con ostomías. Se consideró el índice de concordancia mínimo de 80% para garantizar la validación del material.

**Resultados:**

en cuanto a los objetivos de la guía, todos los jueces evaluaron los ítems como “adecuado” o “totalmente adecuado” con índice de validación de contenido de 1,00. En relación a la estructura y presentación de la guía, el índice fue de 0,84. En el punto atinente a la relevancia, el total fue de 0,97 y el índice general de la guía educativa fue de 0,89, confirmándose la validación junto a los jueces. Todos los ítems de la organización, estilo de escritura, apariencia y motivación del material fueron validados por el público destinatario, alcanzándose el índice de concordancia total de 0,99.

**Conclusión:**

En el contexto de educación en salud, la guía se consideró válida y adecuada para el cuidado de personas con ostomías intestinales, apta para ser utilizada en el ámbito educativo, investigaciones, extensión y en el cuidado a la persona con ostomía intestinal.

## Introduction

Elimination stoma is the name given to an opening created artificially in the abdomen, by surgical procedure, to communicate the internal environment of the intestinal or urinary tract with the external environment, where the elimination of feces and urine occurs. The intestinal stoma can be classified into two types, according to the affected site, subdivided into ileostomy and colostomy^(^ 1 ^)^.

The main causes that lead to the making of a stoma are those of neoplastic origin, which compromise the colon and rectum (colorectal cancer). Estimates from 2018-2019 reveal approximately 582,590 thousand new cases of cancer in Brazil, of which colorectal cancer had an incidence of 37,360 thousand new cases, according to the National Cancer Institute (*Instituto Nacional de Câncer*, INCA)^(^ 2 ^)^. In addition, other causes, such as inflammatory intestinal diseases and abdominal trauma, can also cause the making of a stoma^(^ 3 ^)^.

Living with this condition causes several changes in the life of a person and their family members, which have an impact on physical, psychological and social aspects. The acquisition of a stoma demands new skills for self-care, knowledge about body changes and a new health perspective, as well as coping strategies for a better adaptive process^(^ 4 ^)^.

At this time, the support of family and friends, as well as of the health professionals, is essential for people with stomas. The need for the care provided by the nursing team to this population throughout the perioperative period is emphasized, with the responsibility to provide guidance on the surgical procedure and all stages, ranging from hospitalization, to post-operative care and after discharge from hospital^(^ 5 ^-^ 6 ^)^.

In the post-operative phase, the interventions of the team should be directed towards the realization of self-care, through the resumption of activities of daily living, in addition to particular adjustments and participation in support groups, where the exchange of experiences about living with the stoma and the adaptive process^(^ 7 ^)^.

During the nursing consultations, it is possible to notice several difficulties in self-care, which result in low self-esteem and self-efficacy, associated with the handling and adaptation of the collection equipment, due to complications in the stoma and peristomal area. It is also evident that these people attribute such difficulties to the lack or insufficiency of guidance on the stoma and the necessary care in the pre- and post- operative periods^(^ 8 ^-^ 9 ^)^.

It is necessary, on the part of the nursing team, to establish educational strategies to satisfy both the specific needs of rehabilitation and the improvement of the quality of life of this population^(^ 10 ^)^. In Nursing, health education is a fundamental instrument for a good quality care, as the nurse acts on the teachings of self-care for people with stoma and their families^(^ 11 ^)^.

The increasing use of educational materials (booklet) as resources in health education has assumed an important role in the teaching-learning process, mainly in the therapeutic intervention^(^ 12 ^)^. They are useful for this population, since they favor knowledge, and develop their attitudes, skills and autonomy.

The purpose of health education is to encourage the person’s independence, based on knowledge exchange, in order to encourage self-care and adherence to the necessary treatments^(^ 12 ^)^. Thus, the objective was to validate an educational booklet for people with intestinal stoma as a technological resource in the teaching of self-care.

## Method

A methodological study, developed from October 2016 to November 2017. The research was approved by the Research Ethics Committee of the Federal University of Rio Grande do Norte - (Universidade Federal do Rio Grande do Norte, UFRN) under Certificate of Presentation for Ethical Appreciation (Certificado de Apresentação para Apreciação Ética, CAAE) No. 65942517.9.0000.5537.

For the construction of the educational booklet, it was used as base the results of the integrative literature review and demands reported by people with intestinal stoma treated at the Adult Rehabilitation Center of Rio Grande do Norte, located in Natal/RN.

The review was carried out in the Latin American and Caribbean Health Sciences, Medical Literature Analysis and Retrieval System Online databases, Spanish Health Sciences Bibliographic Index, PubMed Central, Cumulative Index to Nursing and Allied Health Literature, Web of Science and SciVerse Scopus. Articles were selected that suited the inclusion criteria and answered the following guiding question: “What are the main difficulties in caring for people with stoma?” The final sample consisted of 17 articles.

Difficulties in performing self-care are related to stoma cleaning, replacing, leakage, clipping and inadequate bag quality, complications, lack of knowledge about self-care, peristomal skin, discomfort, insecurity, emptying of the bag and irrigation.

Regarding the qualitative study, from which the demands reported by the study population emerged, the sample was composed of 30 people with intestinal stoma seeking care in the stoma therapy sector of the Adult Rehabilitation Center of Rio Grande do Norte (Centro de Reabilitação Adulto do Rio Grande do Norte, CRA-RN) and that met the inclusion criteria. The following guiding question was used: “What were the main difficulties regarding your care with the stoma?”

The results from this stage with people with intestinal stomas are similar to those found in the review, since the main results are related to the problems with the collection bag (replacing and clipping), cleaning, leakage and care with the peristomal skin.

From the general survey of the pertinent contents, teaching sections emerged in which the following were highlighted: the concepts of intestinal stoma, types of stoma, normal stoma characteristics, collection bags, care for the stoma and collection bag and the most frequent doubts of people regarding emptying and replacing the one- and two- piece collector.

Based on these results, dialogues and illustrations were developed to facilitate the understanding of the teachings, even for people with reading difficulties. Also, the assistance of computer programs was obtained, as well as graphic design professionals from the Secretariat of Distance Education (Secretaria de Educação a Distância, SEDIS) and from the Laboratory of Technological Innovation in Health (Laboratório de Inovação Tecnológica em Saúde, LAIS) of the Federal University of Rio Grande do Norte.

These departments assisted in the elaboration of the booklet art according to the theoretical content, previously elaborated, in addition to constructing attractive and easy to understand illustrations. After making the illustrations, the formatting, configuration, and layout of the pages began.

For the validation stage, recommendations on the ideal number of expert judges and the target audience were considered^(^ 13 ^)^. Therefore, nine nurses and 25 people with intestinal stoma participated in the content and appearance validation stage, respectively.

The selection criteria for expert judges were having experience in the area of intestinal stoma, having an article published in the area of interest in an indexed journal or publishing articles involving the stoma theme and having clinical practice in the area of stoma therapy. Content validation makes up the assessment of the universe of information that provides the structure and basis for formulating questions that adequately represent the content^(^ 13 ^)^.

After signing the Free and Informed Consent Form, the characterization questionnaire, the educational material in printed version, and the content validation instrument were sent. To this end, a semi-structured questionnaire^(^ 14 ^)^ was adapted with 17 assertions, organized in a Likert scale format with five judgment options: totally adequate, adequate, partially adequate, not applicable and inadequate. Each statement corresponded to an evaluation item, distributed in three evaluation domains (Objective, Structure and organization, and Relevance). There were also spaces for suggestions and general comments.

After making the necessary adjustments to the booklet, through the suggestions made by the experts, validation with the target audience followed, using an adapted instrument^(^ 13 ^)^, with 13 questions regarding organization, writing style, appearance, and motivation. There were three answer options for each question: positive (yes/easy to understand/clear/interesting), impartial (in part/I don’t know) and negative (no/difficult to understand/confused/uninteresting), according to each type of questioning^(^ 15 ^)^.

This stage consisted of assessing the ease of reading, understanding and appearance, through judgment by the target audience. Twenty-five people with intestinal stoma who attended the CRA, a reference in multiprofessional care for this population across the state, were individually invited.

The inclusion criteria were having an intestinal stoma, aged 18 years or older, attending for care at the CRA during the period proposed for collection and having 10 to 20 minutes to answer the instrument. People with textual and/or visual reading disabilities were excluded, as well as those with impaired mental capacity to perform the assessment of the items.

The final version of the booklet contains 32 pages measuring 150 x 200 mm, printed in the predominant colors of red and orange, on matte A4 paper 150 g/m, secured by staples. And the title of the booklet is “Learning to take care of the intestinal stoma”.

The Content Validity Index (CVI) was used, which measures the agreement of the judges as to the representativeness of the items in relation to the content under study, calculated by dividing the number of judges who evaluated the item as adequate/adequate requiring changes by total of judges (evaluation by item), resulting in the proportion of judges who judged the item valid. To calculate the overall CVI of the instrument, the sum of all CVIs was calculated separately, and divided by the number of items^(^ 16 ^)^. The judges’ suggestions for improving the booklet were analyzed and accepted.

For the validation of the educational booklet by the judges of the area, the items and the instrument as a whole should have CVI greater than or equal to 0.80, for this study. Items with indexes below 0.80 would be excluded or reformulated according to the experts’ suggestions.

For analysis of the items judged by the target audience, data with an agreement level greater than 0.80 for positive responses^(^ 16 ^)^ were also considered validated. The subjects were identified in their statements by the letter “P” followed by an Arabic number from 1 to 25 (P1, P2, P3...), according to the order of their participation in data collection.

## Results

The first version of the educational material submitted for validation by the expert judges was of 32 pages, entitled “Learning to care for the intestinal stoma”. The contents of this booklet contained an initial presentation and, in sequence, the following subjects, which were presented in the form of topics: getting to know the stoma; types of intestinal stomas; normal stoma characteristics; collecting bags; care with the collection bag; frequently asked questions; and, at the end, the bibliographic references consulted to prepare the text.

Regarding the validation of the booklet, nine judges participated in the research, all female, with a degree in Nursing. The mean training time was 8.5 years, minimum of 4 and maximum of 15 years. All had a master’s or doctorate degree, with the theme stoma as the object of study. Five worked in the area of assistance in stoma therapy and the others, in teaching. All had research publications involving the theme stoma and two of them, in addition to this theme, also had publications on instrument validation.

First, the judges evaluated the educational booklet as to the objectives to be achieved with its use. No item was deemed inappropriate or partially adequate or marked as “not applicable”. It was found that, regarding the objectives of the booklet, all items were considered valid, since all the judges classified them as “adequate” or “totally adequate”, which gave a CVI of 1.00 for the proposed objectives, as exposed in Table 1.

**Table 1:** Evaluation of the content judges regarding the objectives of the educational material. Natal, RN, Brazil, 2017

Objectives	Adequate	Totally suitable	CVI*
It is consistent with the needs of people with stomas in relation to self-care	1	8	1.0
Promotes change in behavior and attitudes	4	5	1.0
Can circulate in the scientific community in the stoma area	2	7	1.0

* CVI = Content Validity Index

Subsequently, the judges evaluated the booklet as to its structure and presentation and no item was deemed “inappropriate” or as “not applicable”. It was considered validated, reaching a total CVI of 0.84. However, some were judged to be partially adequate by 22.2% of the judges, as shown in Table 2. These items dealt with the clarity and objectivity of the messages presented; the logical sequence of the proposed content; whether the information was well structured in agreement and spelling; whether the writing style corresponded to the target audience’s level of knowledge; and whether the illustrations were expressive and sufficient.

**Table 2:** Evaluation of the content judges regarding the structure and presentation of the educational material. Natal, RN, Brazil, 2017

Structure and Presentation	Partially Adequate	Adequate	Totally Suitable	CVI*
The educational material is appropriate for guiding people with stoma in relation to self-care.	0	2	7	1.0
The messages are presented in a clear and objective way.	2	3	4	0.77
The information presented is scientifically correct.	1	2	6	0.88
There is a logical sequence of the proposed content.	2	1	6	0.77
The material is appropriate to the socio-cultural level of the proposed target audience.	1	3	5	0.88
The information is well structured in agreement and spelling.	2	4	3	0.77
The writing style corresponds to the level of knowledge of the target audience.	2	3	4	0.77
The information on the cover, back cover, acknowledgments and/or presentation is consistent.	1	1	7	0.88
The illustrations are expressive and sufficient.	2	4	3	0.77
The number of pages is adequate.	1	1	7	0.88
The sizes of the title and topics are appropriate.	1	2	6	0.88

* CVI = Content Validity Index

The analysis of the experts’ comments/suggestions for the content demonstrated the adequacy of the representation of the items and highlighted the necessary modifications. All the expert judges presented some type of comment or suggestion for improving the booklet. For example, word substitutions were suggested to make it easier for everyone to understand.

Regarding the relevance of the educational booklet (Table 3), there were no items judged as “inappropriate” or “not applicable”. Only one judge classified the item “The material proposes to the person with a stoma to acquire knowledge regarding the management of self-care with the stoma” as “partially adequate”. In terms of relevance, the total CVI was 0.97, since the other judges classified all items as “adequate” or “totally adequate”. Consequently, the general CVI of the educational booklet is 0.89, confirming the validation of appearance and content with specialists in the field.

**Table 3:** Evaluation of the content judges regarding the relevance of the educational material. Natal, RN, Brazil, 2017

Relevance	Partially Adequate	Adequate	Totally Suitable	CVI*
The themes portray the key aspects that must be reinforced.	0	1	8	1.00
The material proposes to the person with a stoma to acquire knowledge regarding the management of self-care.	1	3	5	0.88
The material addresses the issues necessary to prevent complications.	0	1	8	1.00
It is suitable for use by any health professional in their educational activities.	0	1	8	1.00

* CVI = Content Validity Index

Regarding validation with the target audience, a total of 25 people with intestinal stoma participated in this stage. They had a minimum age of 18 and a maximum of 66 years old, with a mean of 52 years old, mostly female (64%), married (60%), and 56% with incomplete elementary education. As for the time they lived with the stoma, 52% had between 2 and 10 years of conviviality and 40% had less than 2 years, which shows different times of conviviality so that the person with intestinal stoma acquires greater security and learn to perform your self-care.

As a stage to assess the clarity, understanding and relevance of the content presented in the educational booklet, once the corrections suggested by the judges were made, it was submitted to evaluation by the public with intestinal stoma.

The corrected and printed version of the booklet was delivered individually and, only after the material was handled and read, were they asked to answer the validation instrument, applied by the researcher. Table 4 shows the result of the evaluation of the material by the public with an intestinal stoma.

**Table 4:** Evaluation of people with stoma regarding the organization, style of writing, appearance, and motivation of the booklet. Natal, RN, Brazil, 2017

Items	Positive Answers	Impartial Answers	Agreement Index
**Organization**			
Did the cover catch your attention?	24	1	0.96
Is the content sequence appropriate?	25	0	1
Is the structure of the educational booklet organized?	25	0	1
**Writing style**			
As for the understanding of the phrases, they are: (Easy to understand/Difficult/Do not know)	25	0	1
The written content is: (Clear/Confused/Do not know)	25	0	1
The text is: (Interesting/Uninteresting/Do not know)	25	0	1
**Appearance**			
The illustrations are: (Simple/Complicated/Do not know)	25	0	1
Do the illustrations complement the text?	25	0	1
Do the pages or sections appear organized?	25	0	1
**Motivation**			
In your opinion, anyone with a stoma who reads this booklet will understand what it is about?	25	0	1
Did you feel motivated to read the booklet until the end?	24	1	0.96
Does the educational material address the issues necessary for people with a stoma to perform appropriate care?	25	0	1
Did the educational booklet suggest you act or think about self-care with your stoma?	25	0	1
Overall mean of the agreement index	0.99		

There were no negative answers in the items evaluated by the target audience. All the items of the organization, writing style, appearance, and motivation of the material were considered validated, as they reached a total agreement index of 0.99. Only one answer from the “organization” item, in the question “Does the cover catch your attention?”, was considered an “impartial answer”, and the person with a stoma did not justify the reason why the cover did not fully attract his attention.

Another item that had an impartial answer was about the following question: “Did you feel motivated to read the booklet until the end?” As a justification, it was replied: *I already have a stomafor 5 years and I learned to do everything by myself after a long time, but even so, I am interested in having one to read calmly at home, because I read slowly and reading it all here will cost me more time than it took me to see it page by page* (P12).

**Figure 1:**
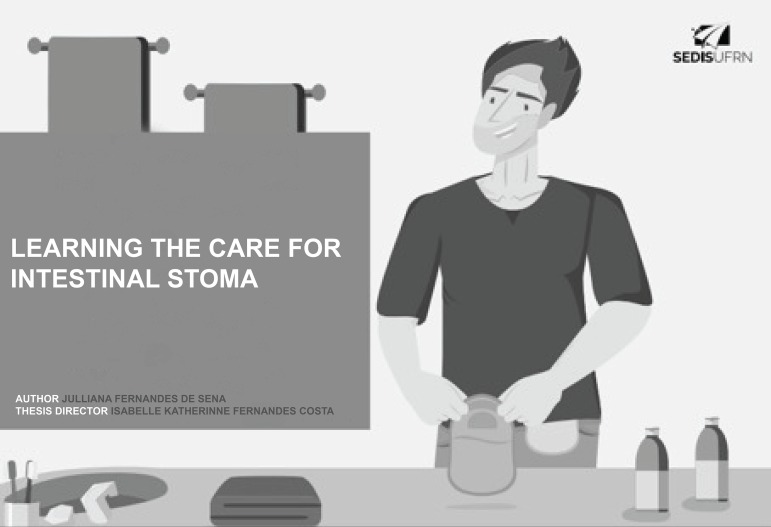
Booklet cover. Natal, RN, Brazil, 2018

After the entire validation process, the educational booklet was completed with 34 pages and started to be offered by professionals in the field. The image of the material cover is shown in Figure 1.

## Discussion

The booklet obtained a general CVI of 0.89 from the judges, showing validity in line with other studies of construction and validation of booklets that obtained CVI > 0.80^(^ 17 ^-^ 19 ^)^. The stage of validation by specialists is essential for the assessment of possible inconsistencies that may impair the understanding of the population for which the booklet is intended, in addition to providing greater methodological rigor in the use of educational technologies^(^ 16 ^)^.

In this process, the participation of stomatherapy judges in the study stands out, since it is a relatively recent specialty in Brazil and exclusive to the professional nurse, which provides contributions to the creation of technologies in stomatherapy area.

This contribution in educational technology validation processes by professional nurses is also anchored in their training and performance characteristics, in which they assume the role of educator of the person with a stoma, of the family, and of the community. It is observed, then, that these professionals have a longer time with this population, which allows to observe more carefully the care needs and strengthen bonds for better interaction and dialogue^(^ 20 ^)^.

In this way, the educational booklet is also seen as a resource to assist professionals in health education, seen as a way of caring to strengthen the capacity and autonomy of others. Thus, health education, as it constitutes a dialogical intervention, allows for continuous training^(^ 12 ^)^, and educational technology in printed format has been widely used to improve knowledge, satisfaction, participation in treatment and self-care for people with a stoma^(^ 21 ^)^.

In a clinical trial conducted in Turkey, educational self-care strategies with slides, educational videos and booklets were implemented in the intervention group, verifying that the self-care scores increased significantly in the intervention group, when compared to the control group (p<0.001), where only routine interventions were established. Education plays an important role in the development of self-care, independence and adaptation for people with stomas^(^ 22 ^)^.

People with stomas who do not receive an adequate health education have deficits in social reintegration and return to the activities of life they performed before surgery. Several studies show that many of these people had a deficient knowledge of stoma and body care, as well as physical and leisure activities, which resulted in social isolation, peristomal complications and impaired daily activities, such as sleep and body hygiene^(^ 23 ^-^ 24 ^)^.

The educational booklet is an important tool in providing educational support to this population, as it addresses aspects of stoma care, bag replacing, hygiene, clothing and when and where to seek professional help, in order to encourage autonomy for the development of the self-care.

Access to this material will contribute to the acquisition of knowledge that will help people with intestinal stoma in the process of adapting to the new life condition, in the resignification of their self-image and self-concept, overcoming fears, as well as taboos arising from altering of body image^(^ 12 ^)^.

Thus, the information in the booklet seeks to achieve basic knowledge on the subject, both from the theoretical content and from the illustrations. Therefore, there was a concern that these were explanatory and complementary in relation to teaching in written form, facilitating visual communication and the approximation with knowledge by the subjects with limited approximation with the written language^(^ 17 ^-^ 18 ^)^.

Among these aspects to be considered in communication for teaching, it is important to highlight the adequacy of language to this target audience, facilitating the understanding by people with varying levels of education^(^ 17 ^-^ 18 ^)^.

In line with these aspects, the target audience evaluated the booklet in a positive way, considering it important, very useful, adequate and explanatory, especially for people with little stoma time who do not yet have the knowledge about the skills they should develop to ensure self-care, enabling better adaptation and prevention of future complications. This evaluation was important to validate the material and disseminate information for daily care with the stoma and the use of the collection bag.

The provision of educational material helps and standardizes the guidelines to be carried out, in addition to serving for consultation of the target audience aiming for health care. The educational booklet is an instrument that contributes to care, especially in the period immediately after the making of the stoma, since during hospitalization it becomes difficult to assimilate so much new information^(^ 8 ^)^.

A study conducted in the United States with newly stomized people agrees that establishing a bridge between the transition period and hospital discharge and the initial clinical follow-up, using sensitive, educational and timely interventions, should be a priority in this population. Health professionals should strive to help these patients return to as close as possible to their normal function^(^ 25 ^)^.

In this perspective, the educational booklet plays an important role, as this population lacks educational materials of this nature that can help people with stoma, the family member, and the caregiver^(^ 26 ^)^. In addition, there is a shortage of publications focused on nursing care and technologies, mainly directed to the health education process^(^ 26 ^-^ 27 ^)^.

The presentation of educational materials developed by professionals must have wide diffusion and dissemination, in order to collaborate with the promotion of health education to assist in the development of self-care and improvement in the individual’s quality of life^(^ 22 ^)^. Therefore, it is believed that the construction and validation of this educational booklet will contribute to adherence to the self-care of this population.

Printed or digital educational material, available in PDF (Portable Document Format), has been used by health professionals at ARC as a teaching tool. For this reason, the participatory approach used in the construction and validation of this educational material allowed to identify the needs of people with intestinal stoma, which indicate the content of the booklet as corresponding to their own demands.

As a limitation of the study, there is the high cost of the printed material to be made available to the target population, as well as the difficulty of using the online booklet by people who do not have access to this resource. In addition, people with a low cognitive impairment and dementia will find it difficult to assimilate the information contained in the material.

Thus, new validation studies with audiovisual technologies are suggested to overcome these limitations and assist this population in the care of the stoma. As well as adding technologies that can be reproduced and disseminated, in order to assist in the scientific advancement and health of this population.

## Conclusion

The research promoted the validation of the educational booklet “Learning to care for the intestinal stoma”, being validated in terms of the objective, structure, organization and relevance domains by the specialists and in terms of organization, writing style, appearance and motivation with the target population, in which all obtained CVI greater than 0.80.

Thus, in the context of health education, the booklet was considered valid and suitable for the care of intestinal stomas and could be used in teaching, research, extension and clinical care environments. The booklet can assist in the autonomy and self-care of people with stomas, as well as in supporting professionals in assisting this population.
